# UBXN1 promotes liver tumorigenesis by regulating mitochondrial homeostasis

**DOI:** 10.1186/s12967-024-05208-5

**Published:** 2024-05-21

**Authors:** Kun Jiao, Guiqin Xu, Yun Liu, Zhaojuan Yang, Lvzhu Xiang, Zehong Chen, Chen Xu, You Zuo, Zhibai Wu, Ningqian Zheng, Wangjie Xu, Li Zhang, Yongzhong Liu

**Affiliations:** 1 State Key Laboratory of Systems Medicine for Cancer, Shanghai Cancer Institute, Renji Hospital, School of Biomedical Engineering, Shanghai Jiao Tong University, Shanghai, 200240 China; 2 State Key Laboratory of Systems Medicine for Cancer, Shanghai Cancer Institute, Renji Hospital, School of Medicine, Shanghai Jiao Tong University, Shanghai, 200240 China; 3 Laboratory Animal Center, Instrumental Analysis Center, Shanghai Jiao Tong University, Shanghai, 200240 China

**Keywords:** UBXN1, Hepatocellular carcinoma, PHB, Mitochondrial homeostasis, Apoptosis

## Abstract

**Background:**

The maintenance of mitochondrial homeostasis is critical for tumor initiation and malignant progression because it increases tumor cell survival and growth. The molecular events controlling mitochondrial integrity that facilitate the development of hepatocellular carcinoma (HCC) remain unclear. Here, we report that UBX domain-containing protein 1 (UBXN1) hyperactivation is essential for mitochondrial homeostasis and liver tumorigenesis.

**Methods:**

Oncogene-induced mouse liver tumor models were generated with the Sleeping Beauty (SB) transposon delivery system. Assessment of HCC cell growth in vivo and in vitro, including tumour formation, colony formation, TUNEL and FACS assays, was conducted to determine the effects of UBXN1 on HCC cells, as well as the involvement of the UBXN1-prohibitin (PHB) interaction in mitochondrial function. Coimmunoprecipitation (Co-IP) was used to assess the interaction between UBXN1 and PHB. Liver hepatocellular carcinoma (LIHC) datasets and HCC patient samples were used to assess the expression of UBXN1.

**Results:**

UBXN1 expression is commonly upregulated in human HCCs and mouse liver tumors and is associated with poor overall survival in HCC patients. UBXN1 facilitates the growth of human HCC cells and promotes mouse liver tumorigenesis driven by the *NRas*/*c-Myc* or *c-Myc*/sh*p53* combination. UBXN1 interacts with the inner mitochondrial membrane protein PHB and sustains PHB expression. UBXN1 inhibition triggers mitochondrial damage and liver tumor cell apoptosis.

**Conclusions:**

UBXN1 interacts with PHB and promotes mitochondrial homeostasis during liver tumorigenesis.

**Supplementary Information:**

The online version contains supplementary material available at 10.1186/s12967-024-05208-5.

## Background

HCC is the third leading cause of cancer-related mortality worldwide, with a 5-year survival rate of approximately 18% [1, 2]. The incidence of genetic alterations in HCCs is relatively low compared to that in other cancer types, and alterations only occur in some subgroups of HCCs; aberrant molecular events regulating the processes of apoptosis and mitochondrial homeostasis are commonly observed in HCC and drive hepatocarcinogenesis [3–6].

The maintenance of mitochondrial homeostasis in tumor cells is critical for cellular energy production and the survival of tumor cells under genetic or environmental stresses [3, 4, 7]. Oncogenic driver mutations, such as those affecting *KRas* and *c-Myc*, maintain proper mitochondrial bioenergetics and ATP levels by modulating the expression of mitochondrial proteins, including MFN2, OPA1 and DRP1, in transformed cells [4, 8–10]. Loss of these mitochondrial proteins damages mitochondria and shifts mitochondrial dynamics, which alters mitochondrial membrane potential and oxidative phosphorylation, resulting in excessive production of reactive oxygen species (ROS) and induction of mitochondria-associated apoptosis, which are detrimental to liver tumorigenesis [3–5, 11, 12]. Notably, the integrity of mitochondria is tightly regulated by mitochondrial inner membrane proteins, such as PHB, TIMM13 and VDAC1, which are involved in membrane architecture and dynamics [11, 13–16]. Emerging evidence has shown that PHB is highly expressed in multiple types of cancer, including pancreatic cancer (PDAC), breast carcinoma (BC) and gastric cancer (GC), and participates in biological processes related to tumorigenesis [17–19]. For instance, PHB is phosphorylated by AKT at Thr258 and thereby facilitates bladder cancer cell proliferation [20]. Additionally, PHB promotes the growth and metastasis of cervical cancer through binding to Raf-1 and enhances the Ras-mediated activation of the Raf/MEK/ERK pathway [21]. Targeting PHB using genetic or pharmacological approaches decrease the expression of Bcl-2, activates caspase(s) and induces mitochondria-dependent apoptosis [22, 23]. However, the molecular events controlling PHB expression in tumor development and progression remain elusive.

UBXN1, a member of the ubiquitin regulatory X domain-containing protein (UBXN) family, comprises 13 members that share similar functional ubiquitin-associated domain (UBA) or ubiquitin regulatory X (UBX) domains and is implicated in regulating multiple cellular processes, including the innate immune response, replisome disassembly and mitophagy [24–28]. UBXN1 has dual effects on tumor growth in different types of cancer [29–31]. YTHDF2 recognizes METTL3-mediated m^6^A on UBXN1 mRNA and decreases its expression, which in turn promotes NF-κB signaling and the malignant progression of gliomas [31]. In contrast, the inhibition of UBXN1 attenuated prostate cancer cell proliferation by inducing mitochondria-associated apoptosis [29]. Furthermore, UBXN1 recognizes and binds to autoubiquitinated BRCA1, a tumor suppressor for breast cancer [32], and inhibits its enzymatic function, suggesting that UBXN1 plays an oncogenic role in cancer.

In this study, we found that the expression of UBXN1 was upregulated in *NRas* G12V/*c-Myc*-driven and *c-Myc*/sh*p53*-driven mouse HCC tumors and human HCCs. UBXN1 maintained PHB expression by binding to it, which promoted mitochondrial homeostasis. Aberrant upregulation of UBXN1 inhibited mitochondria-associated cellular dysfunction and promoted liver tumorigenesis.

## Materials and methods

### Cell lines and tissue specimens

PLC/PRF/5, Hepa1-6 and HEK-293T cell lines were purchased from American Type Culture Collection (ATCC, Manassas VA, USA). HCC cell line MHCC97H was supplied by Liver Cancer Institute, Fudan University (Shanghai, China). Mouse LPC-HRas cells were previously generated in our laboratory [33]. All cells were cultured in DMEM (Gibco) containing 10% FBS, 50 U/ml of penicillin and streptomycin (Yeasen, China) at 37 °C under 5% CO2. The absence of mycoplasma contamination was determined by PCR assay. Human 21 paired HCC samples were obtained from HCC patients who received curative surgery in the Department of Liver Surgery, Renji Hospital, School of Medicine, Shanghai Jiaotong University (Shanghai, China). The tissue microarray LVC-1605 is from Shanghai Outdo Biotech Company (Shanghai, China). The clinical information of HCC patients is presented in supplementary Tables 4 or 5.

### Reagents and antibodies

Puromycin (CL13900) was purchased from MCE. CCCP (C2759) and MTT (Thiazolyl Blue Tetrazolium Bromide, M5655) were purchased from Sigma. Lipofectamine 2000 Transfection Reagent was purchased from Invitrogen. Polyethyleneimine transfection reagent (23966-1) was purchased from Polysciences. JC-1 fluorescent probe (IJ0300) was purchased from Solarbio. Caspase Inhibitor Z-VAD-FMK (C1202) and TUNEL Apoptosis Assay Kit (C1089) were obtained from Beyotime. MitoTracker™ Deep Red FM (M22426) and MitoTracker™ Green FM (M7514) Fluorescent Dyes were from ThermoFisher. DMSO was from SantaCruz. All antibodies used in this study are listed in Supplementary Table 1.

### Animal studies

Male C57BL/6 mice (6–8 weeks old) were purchased from Lingchang Biotechnology Co. (Shanghai, China). BALB/c nude mice were purchased from Shanghai SLAC Laboratory Animal Company (Shanghai, China). C57BL/6 mice were subjected to hydrodynamic tail vein injection (HDTVi) with the endotoxin-free plasmid combination [28]: (1) 2.5 µg SB100, 12 µg pT-Caggs-*NRas* G12V, 12 µg pT3-EF1α-*c-Myc*; (2) 2.5 µg SB100, 12 µg pT2-sh*p53*, 12 µg pT3-EF1α-*c-Myc*; (3) 2.5 µg SB100, 12 µg pT-Caggs-*NRas*G12V, 12 µg pT3-EF1α-*c-Myc*, 12 µg plentiV2- *Cas9*-sg*Ubxn1* (or sg*Scramble*); (4) 2.5 µg SB100, 12 µg pT2-sh*p53*, 12 µg pT3-EF1α-*c-Myc*, 12 µg plentiV2- *Cas9*-sg*Ubxn1* (or sg*Scramble*); (5) 2.5 µg SB100, 12 µg pT-Caggs-*NRas*G12V, 12 µg pT3-EF1α-*c-Myc*-IRES-PHB (or Control), 12 µg plentiV2- *Cas9*-sg*Ubxn1* (or sg*Scramble*). All mice were sacrificed when they developed abdominal swelling and liver weights and other data were recorded, and the livers were removed for further study. Six-week-old BALB/c nude mice were subcutaneously injected with MHCC97H cells (7 × 10^6^) utilizing shRNA knockdown of UBXN1 or control. Mice were subcutaneously injected with MHCC97H cells (5 × 10^6^) expressing UBXN1 or vector control. Once the tumors were formed, the tumor size of the mice was measured and recorded every two days. After the mice reached a tumor size of 1000 mm^3^, the mice were sacrificed to remove the tumors for further study. Mice were housed in a SPF condition. All experiments were conducted in accordance with National Institutes of Health guidelines and were agreed by the Institutional Animal Care and Use Committee of Shanghai Jiao Tong University and Shanghai Cancer Institute.

### Plasmids and stable cell line construction

V5-tagged UBXN1 (full length), UBXN1 with the deletion of N-terminal UBA domain (ΔUBA: 1-42aa) and the deletion of C-terminal UBX domain (ΔUBX: 205-313aa) were cloned into PLVX-ZSgreen plasmid. The overexpression plasmids (UBXN1-Flag, PHB-His, PHB2-Flag and GFP-UBXN1) were cloned into PLVX-IRES-Puro. The shRNA sequence was inserted into pLKO.1. The *UBXN1* gDNA sequence was inserted into LentiV2-CRISPR plasmid. HEK293T cells and packaging plasmids (psPAX2, pMD2.G) were used for lentivirus production. HCC cells were infected with lentivirus adding polybrene. Cells overexpressing the PLVX-ZSgreen plasmid were sorted by flow cytometry. SB100, pT-Caggs-*NRas* G12V, pT3-EF1α-*c-Myc* and pT2-sh*p53* plasmids were from Addgene. pT3-EF1α-*c-Myc*-IRES-PHB palasmid was self-designed and constructed by homologous recombination using pT3-EF1α-*c-Myc* as a template. All plasmids injected into the tail vein of mice were extracted using an endotoxin-free plasmid extraction kit (DP117, TIANGEN). All sequences of designed shRNA or guide DNA in this study are listed in Supplementary Table 2.

### Western blotting and Co-IP

Western blotting and IP assay were conducted following the protocols [34]. In brief, the cells or tissues were lysed in RIPA lysis buffer (Beyotime, P0013B) with Protease Inhibitor Cocktail (Roche, 05892970001) and Phosphatase Inhibitor (Roche, 04906845001) for 1 h. After 12,000 rpm centrifugation for 20 min at 4 °C, the supernatant was taken. Then, the protein supernatants were separated using SDS-PAGE and transformed onto nitrocellulose filter membranes (Pall Corporation, 66485). After blocking by 5% BSA for 1 h, the membranes were incubated with primary antibodies at 4 ℃ overnight. The blots were measured by laser scanner (Odyssey, Licor, USA). Cells were lysed in western blot & IP lysis buffer (Beyotime, P0013). The lysates were incubated with protein A/G beads (Santa Cruz, sc-2002) and primary antibody for 12 h at 4 °C. The precipitates were collected by centrifugation at 1500 rpm for 5 min at 4 °C and washed three times with the cold 1×TBS. The protein samples were subjected to western blotting analysis.

### Mass spectrometry analysis

The protein samples were preprocessed with reference to protocols in the published article [34]. Briefly, cell lysates were immunoprecipitated and separated by SDS-PAGE, then stained with Caumas Brilliant Blue. LC-MS/MS analysis and identification of peptide mixtures were performed by Hangzhou PTMBIO Biotechnology Co.

### Quantitative real-time PCR

The qRT-PCR assay referenced the protocols of the published article [34]. The mRNA from the cells were extracted by RNAiso Plus (Takara, 9108) based on the manufacturer’s manual. Then, the mRNA was reverse transcribed into cDNA by Hifair™ III Super Mix plus (yeasen, 11141C). qRT-PCR was conducted using Hieff® qPCR SYBR Green Master Mix (yeasen, 11202ES03) and the Applied Biosystems 7500 (Life Technologies, USA). The gene expression was normalized with GAPDH. All sequences of designed primers in this study are listed in Supplementary Table 3.

### RNA-sequencing (RNA-seq) and bioinformatics analysis

RNA from tumor cell lines was extracted with TRIZOL Reagent (Life Technologies, USA) following the manufacturer’s instructions. Briefly, transcriptome library was built by the TruSeq™ RNA preparation kit (Illumina, CA). Paired-end library was conducted by Illumina NovaSeq 6000 system. The reads were mapped to the human reference genome (GRCh38) for human cells, using Hisat2 to produce the BAM files. The expression level of transcripts was calculated according to the per Transcripts Per Million reads (TPM) method. Differentially expressed genes were estimated by limma algorithm. The pathway enrichment analysis, Kyoto Encyclopedia of Genes and Genomes (KEGG), was performed using the Cluster Profiler package through R programming language.

The GSE14520 transcriptome dataset was downloaded from the GEO database. The TCGA-LIHC gene expression dataset was downloaded from the TCGA data portal. The ICGC-LIHC dataset was obtained from the ICGC data portal. The survival and survminer packages were used to generate Kaplan‒Meier plots. The cut-off value for *UBXN1* expression was determined by quartile. The tumor samples from the TCGA-LIHC database were classified into two groups based on the transcript levels of *UBXN1*: the top 25% were classified as the high expression group, and the bottom 25% were classified as the low expression group. GSEA was conducted using GSEA version 4.2.3 of the Broad Institute. A false discovery rate (FDR) < 0.05 and *p* value < 0.05 were considered to indicate a statistically significant difference. The raw RNA-seq data and detailed experimental design are deposited under GSE247383.

Using the RNA-seq data of MHCC97H cells, the genes regulated by sh*UBXN1* (sh*UBXN1* vs. shRFP, fold change ≥ 2 or ≤ 0.5, *p* value < 0.05.) were identified and subjected to KEGG enrichment analysis via the clusterProfiler package.

### MTT and Colony formation assay

Cells were spread on a 96-well plate at a density of 1000 cells per well. At the indicated time points, 15 µL of MTT (0.5 mg/ml) was added to each well, followed by incubation at 37 °C for 3 h. Absorbance values at 493 nm were detected using a Fluorescence Microplate Reader (Infinite ^TM^ M200, TECAN). Cells were seeded in 6-well plates at a density of 2000 cells per well. After 10 days, cells were fixed in methanol and stained with crystal violet (Beyotime, C0121).

### Flow cytometric analysis

Apoptotic cells were detected by using APC-Annexin V (Biolegend, 640920) and 7-AAD (Biolegend, 640926) Apoptosis Detection Kit. The fluorescent probe MitoTracker Deep Red (50nM) was used to label mitochondria. In addition, mitochondrial membrane potential changes were detected using the JC-1(5µM) Assay Kit. All these experiments were performed according to the corresponding manufacturer’s instructions. At least 5000 live cells were detected by flow cytometric analysis on a FACS flow cytometer (Becton Dickinson, LSRFortessa). Experimental data were analyzed using Flow Jo software v10.2.

### Immunohistochemistry

Tumors or liver tissues were fixed and embedded in paraffin. Tissue sections were deparaffinized with xylene and rehydrated in decreasing concentrations of alcohol, followed by immersion in 3% hydrogen peroxide for 10 min at room temperature. Then, the sections were boiled in 0.01 mol/L citrate buffer for 20 min on low heat for antigen extraction. The tissue sections were then cooled to room temperature and blocked with immunostaining blocking solution for 1 h. The tissue sections were then incubated with primary antibody at 4 °C overnight. The sections were rinsed three times with PBS and incubated with secondary antibody at room temperature for 1 h. After rinsing three times with PBS, the sections were stained with hematoxylin for nuclear staining and dehydrated with different concentrations of ethanol.

### Immunofluorescence

Cells were fixed with 4% paraformaldehyde for 20 min at room temperature, and permeabilized with 0.1% Triton X-100 for 20 min. Cells were then blocked with immunostaining blocking solution (Beyotime, P0023A) for 1 h and incubated with primary antibody at 4 °C overnight. The cells were washed three times with PBS and incubated with secondary antibody at room temperature for 1 h. After washing three times with PBS, the cells were stained with DAPI for 15 min. The fluorescent probe Mito Tracker Green (50nM) was used to label mitochondria of living cells. The experiment was performed according to the corresponding manufacturer’s instructions. Pictures of cells at 488 nm were taken using a fluorescence microscope (TCS SP8, Leica).

### TUNEL assay

Fragmented DNA in cells was labelled using a TUNEL kit with red fluorescence according to the manufacturer’s instructions. In brief, cells were fixed with 4% paraformaldehyde for 20 min at room temperature. The DNA fragments of cells were labelled with dUTP by the terminal transferase reaction. Cell nuclei were stained with DAPI. Pictures of cells or tissues at 550 nm were taken using a fluorescence microscope (TCS SP8, Leica).

### Mitochondrial DNA quantification

The mtDNA extraction kit (Abcam, ab65321) was used to extract DNA from the cells according to the corresponding manufacturer’s instructions. The DNA then analyzed by qPCR using primers targeting mtDNA (tRNA-Leu). The mtDNA primers were synthesized to detect tRNA-Leu (Forward: CACCCAAGAACAGGGTTTGT; Reverse: TGGCCATGGGTATGTT GTTA) and GAPDH.

### Statistical analysis

All experiments were repeated at least three times and data are expressed as mean ± SD. Statistical analysis was performed using GraphPad Prism7. Paired and unpaired and two-tailed Student’s t-tests were used to compare the data between the two groups. Survival analysis was performed using the log-rank test. Pearson’s test was used to measure correlation between the two groups. *p < 0.05; **p < 0.01, and ***p < 0.001. All results with *p* values < 0.05 were statistically significant.

## Results

### UBXN1 is aberrantly upregulated in HCC and associated with poor outcomes

**Fig. 1:**
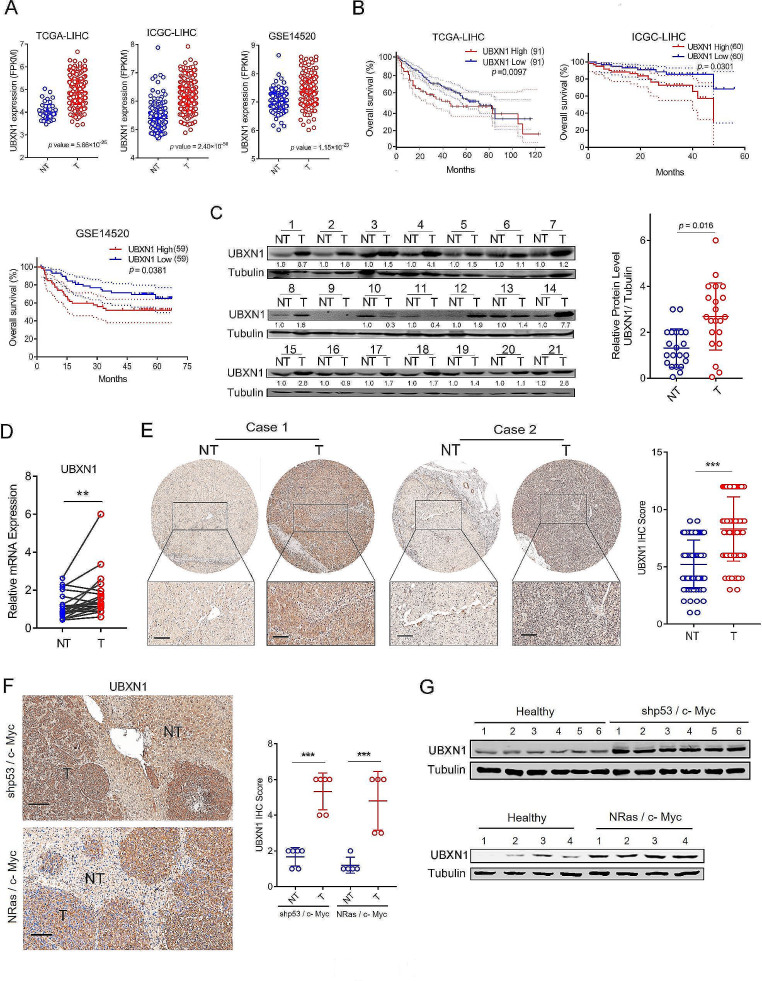
UBXN1 expression is upregulated in HCC and correlated with poor clinical prognosis. (**A**) The expression level of *UBXN1* (FPKM) in HCC (T) and nontumor (NT) tissues from TCGA-LIHC (NT = 50, T = 374), ICGC-LIHC (NT = 202, T = 243) or GSE14520 (NT = 241, T = 247) databases. (**B**) Kaplan–Meier survival curves showing overall survival of HCC patients stratified by UBXN1 mRNA expression level (TCGA-LIHC (*n* = 364), ICGC-LIHC (*n* = 243) or GSE14520 (*n* = 242). (**C**) Western blotting analysis of UBXN1 in 21 paired clinical HCC tissues (T) and adjacent normal tissues (NT). The quantitative measurement was conducted to analyze grayscale value (right, UBXN1/Tubulin, paired Mann Whitney test). (**D**) qRT-PCR analysis of UBXN1 in 21 paired clinical HCC tissues (T) and adjacent normal tissues (NT). (**E**) Representative IHC images (left) and IHC score (right) of UBXN1 expression in paired clinical HCC tissues (T) and adjacent normal tissues (NT), *n* = 80. Scale bar: 100 μm. (**F-G**) The C57BL/6 mice were hydrodynamically injected with indicated plasmids (*n* = 5 or 6). IHC images (**F**, left) and IHC score (**F**, right) of mouse livers at 4 or 7 weeks post hydrodynamic injection. Scale bar: 100 μm. Western blotting analysis of UBXN1 expression in liver tissues (**G**). The data are presented as mean ± SD. **, *p <* 0.01; ***, *p <* 0.001; by two-tailed Student t test

To address the potential involvement of UBXN proteins in the development and progression of HCC, we first analyzed the mRNA expression patterns of UBXN proteins in the tumor and nontumor tissues of HCC patients and found that the transcript level of *UBXN1* was consistently and significantly elevated in tumor tissues compared to nontumor tissues from the TCGA-LIHC, ICGC-LIHC and GEO14520 cohorts (Fig. 1A and Supplementary 1A–C). Furthermore, survival analysis revealed that patients with high *UBXN1* levels had shorter overall survival than those with low *UBXN1* levels (Fig. 1B). To support the *in silico* results, we assessed the expression profiles of UBXN1 in 21 HCC clinical specimens and observed that UBXN1 protein expression was robustly upregulated in ∼ 61.9% of the tumor tissue samples, as evidenced by the increased protein and mRNA levels in the tumor tissue samples compared with nontumor tissue specimens. (Fig. 1C-D). Additionally, IHC analysis of UBXN1 expression in 80 paired HCC patient samples revealed that the UBXN1 protein was predominantly located in the cytoplasm and highly expressed in HCC cells compared with adjacent cells (Fig. 1E). Next, we measured expression of UBXN1 in mouse liver tumors by using two set of oncogenic-driven primary liver cancer model, which were generated by the SB transposon delivery of the plasmids expressing *NRas* G12V (*NRas*)/*c-Myc* and *c-Myc*/sh*p53*, and developed HCC approximately 2 or 5 weeks post-HDTVi, respectively (Supplementary 1D). After confirming the efficiency of *NRas*- and *c-Myc*-overexpression as well as *p53*-knockdown (Supplementary 1E), we found that the protein abundance of UBXN1 was aberrantly increased in mouse liver tumors induced by *NRas* G12V/*c-Myc* or *c-Myc*/sh*p53* (Fig. 1F–G). Collectively, these results suggest that UBXN1 may contribute to the development and progression of HCC.

### UBXN1 promotes oncogene-driven liver tumorigenesis

**Fig. 2:**
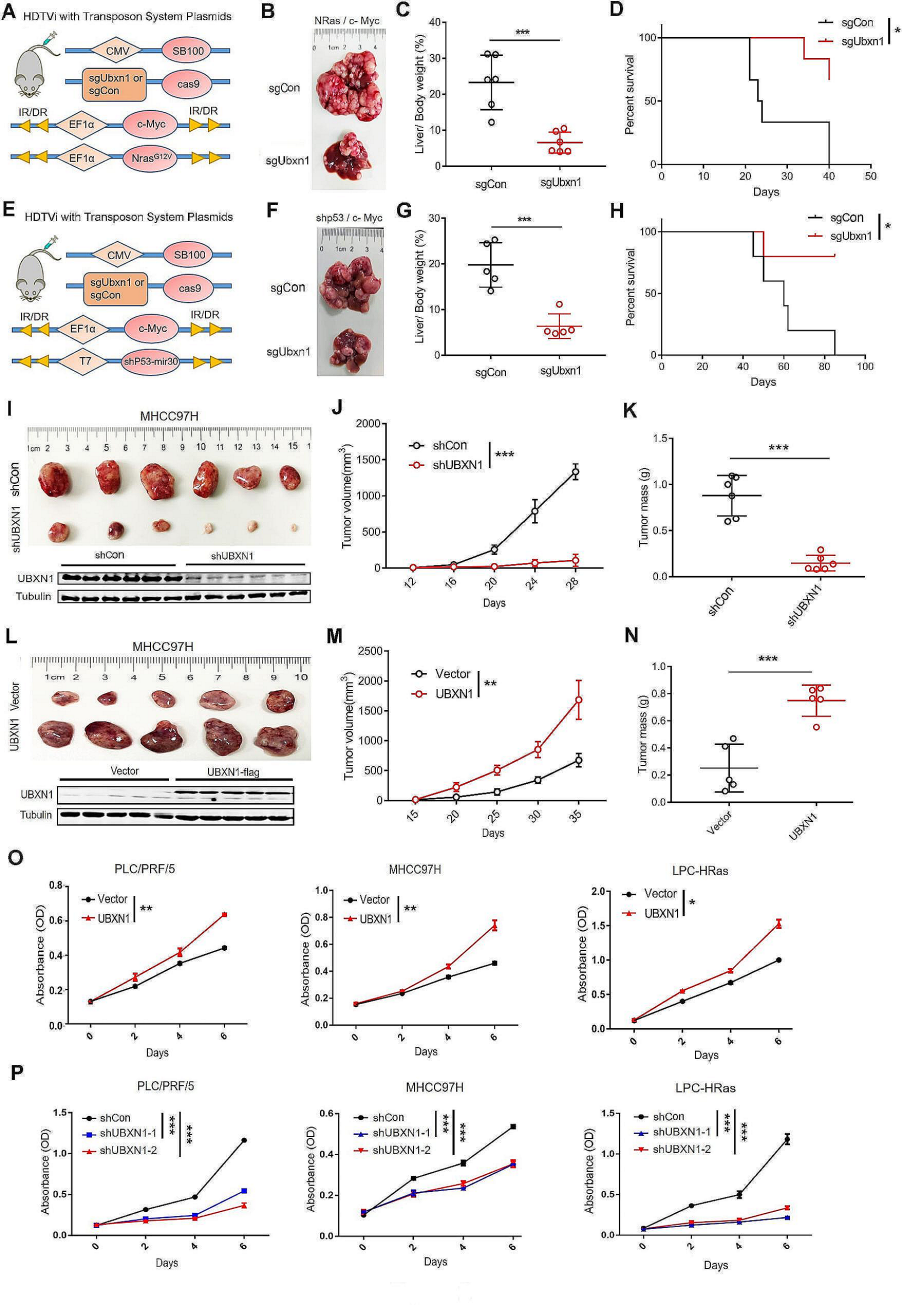
UBXN1 promotes HCC cell proliferation and tumorigenicity. (**A**–**D**) The C57BL/6 mice were hydrodynamic co-injected transposon-based vectors expressing *NRas* G12V, *c-Myc*, along with vector expressing Cas9 and a CRISPR guide RNA targeting *UBXN1* or *scramble RNA* (*n* = 6). Schematic representation of the HDTVi (**A**), morphology of livers (**B**), ratio of liver/body weight (**C**) or Kaplan-Meier overall survival curves (**D**) were shown. (**E**–**H**) The indicated vectors were hydrodynamic injected into C57BL/6 mice (*n* = 5). Schematic of vectors (**E**), morphology (**F**), ratio of liver/body weight (**G**) or Kaplan-Meier overall survival curves (**H**) were shown. (**I**–**K**) BALB/C nude mice were subcutaneously implanted with 7 × 10^6^ UBXN1-knockdown or control MHCC97H cells (*n* = 6). Tumors were harvested at day 28 post implantation. Representative pictures (**I**), tumor volumes (**J**) and tumor mass (**K**) were shown. (**L**–**N**) BALB/C nude mice were subcutaneously implanted with 5 × 10^6^ UBXN1-ovexpressed and control MHCC97H cells (*n* = 5). Tumors were harvested at day 35 post implantation. Representative pictures (**L**), tumor volumes (**M**) and tumor mass (**N**) were shown. (**O**) The indicated cells were cultured with medium containing 0.1% FBS with starvation for one day or two, and then switched to complete medium for the following days. The growth capacity was tested by MTT assay. (**P**) MTT assay was used for determine the growth capacity of indicated cells. The data are presented as mean ± SD. **, *p <* 0.01; ***, *p <* 0.001 by two-tailed Student t test

To explore the requirement of UBXN1 for liver tumorigenesis, we transduced CRISPR/*Cas9* vectors targeting *Ubxn1* together with transposon plasmids expressing *NRas*/*c-Myc* or *c-Myc*/sh*p53* into C57BL/6 mice by HDTVi (Fig. 2A–H). The knockout efficiency of *Ubxn1* with the designed sgRNA was confirmed prior to the experiments (Supplementary 2A). As expected, knockout of *Ubxn1* significantly inhibited tumor development in these two sets of autochthonous liver tumor models, as evidenced by a decreased liver/weight ratio and increased survival in the *Ubxn1*-KO (*sgUbxn1*) group compared with those in the control (*sgCon*) group (Fig. 2B–D and F–H). Consistent with these findings, xenograft experiments revealed substantial decreases in the volume and weight of UBXN1-knockdown human HCC tumors compared with control tumors (Fig. 2I–K). Notably, UBXN1 overexpression had the opposite effect on the growth of HCC tumors (Fig. 2L–N). Next, we investigated whether UBXN1 regulates tumor proliferation in vitro. The results showed that overexpression of UBXN1 strongly promoted the proliferation potential of tumor cells cultured under serum starvation conditions, and that knockdown of UBXN1 suppressed the proliferation of cultured PLC/PRF/5, MHCC97 H and LPC-HRas cells (Fig. 2O-P). A colony formation assay revealed similar results: knockdown of UBXN1 inhibited clonogenicity, whereas ectopic UBXN1 expression increased clonogenicity in cells cultured in serum-insufficient conditions (Supplementary 2B-C), suggesting that UBXN1 is required for liver tumor growth.

### UBXN1 promotes tumor cell survival and growth by inhibiting cell apoptosis

**Fig. 3:**
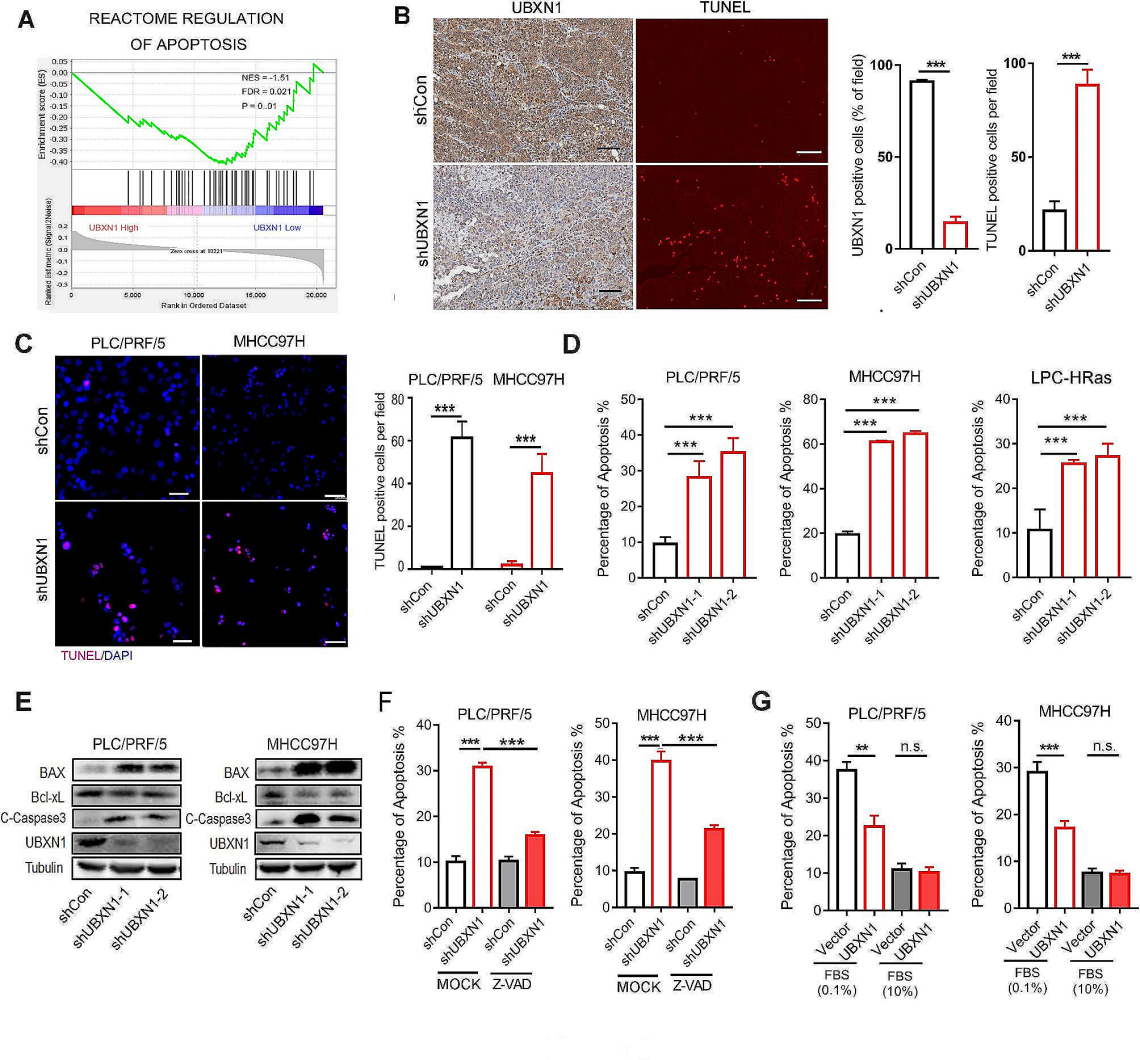
Inhibition of UBXN1 induces HCC cell apoptosis. (**A**) GSEA of TCGA-LIHC dataset with UBXN1 transcript levels and REACTOME _REGULATION_OF_APOPTOSIS signature. (**B**) UBXN1 staining (left) and TUNEL staining (right) of indicated samples derived from MHCC97H xenograft tumor. Scale bar: 100 μm. (**C**) IF staining of TUNEL in indicated PLC/PRF/5 and MHCC97H cells. Scale bar: 100 μm. (**D**) Flow cytometry analysis of apoptotic ratio in PLC/PRF/5, MHCC97H and LPC-HRas cells. (**E**) Western blotting analysis of indicated proteins in UBXN1-knockdown PLC/PRF/5 and MHCC97H cells. (**F**) Flow cytometry analysis of apoptosis ratio in indicated cells treated with or without Z-VAD-FMK (20µM) for 48 h. (**G**) Flow cytometry analysis of apoptosis ratio in indicated PLC/PRF/5 and MHCC97H cells coculturing with or without medium containing 0.1%FBS for 48 h serum starvation. The data are presented as mean ± SD. ***, *p <* 0.001 by two-tailed Student t test

We next performed GSEA of the genes differentially expressed in TCGA-LIHC tumors with a high abundance of *UBXN1* transcripts (UBXN1^high^) compared with those in the UBXN1^low^ group and revealed a significant correlation between the mRNA levels of *UBXN1* and genes involved in the regulation of apoptosis (Fig. 3A). We further used a TUNEL assay to examine tumor cell apoptosis in human HCC xenografts, and the results showed that UBXN1-knockdown greatly increased the number of TUNEL^+^ cells (Fig. 3B). Additionally, the number of TUNEL^+^ apoptotic cells was increased in cultured PLC/PRF/5 and MHCC97H cells upon UBXN1-knockdown (Fig. 3C). FACS analysis also revealed that the percentages of apoptotic cells resulting from UBXN1-knockdown were robustly greater than those of control cells (PLC/PRF/5, MHCC97H, and LPC-HRas, Fig. 3D). These results were further corroborated by increased levels of BAX and cleaved caspase-3 and decreased expression of Bcl-xL in UBXN1-knockdown HCC cells (Fig. 3E). Furthermore, the induction of cell apoptosis by UBXN1-knockdown was efficiently reversed by the pan-caspase inhibitor Z-VAD (Fig. 3F). Notably, UBXN1 overexpression markedly attenuated tumor cell apoptosis induced by serum starvation (Fig. 3G). These results suggest that UBXN1 promotes HCC cell survival and tumor growth by inhibiting caspase-mediated cellular apoptosis.

### UBXN1 alleviates mitochondrial damage in HCC cells

**Fig. 4:**
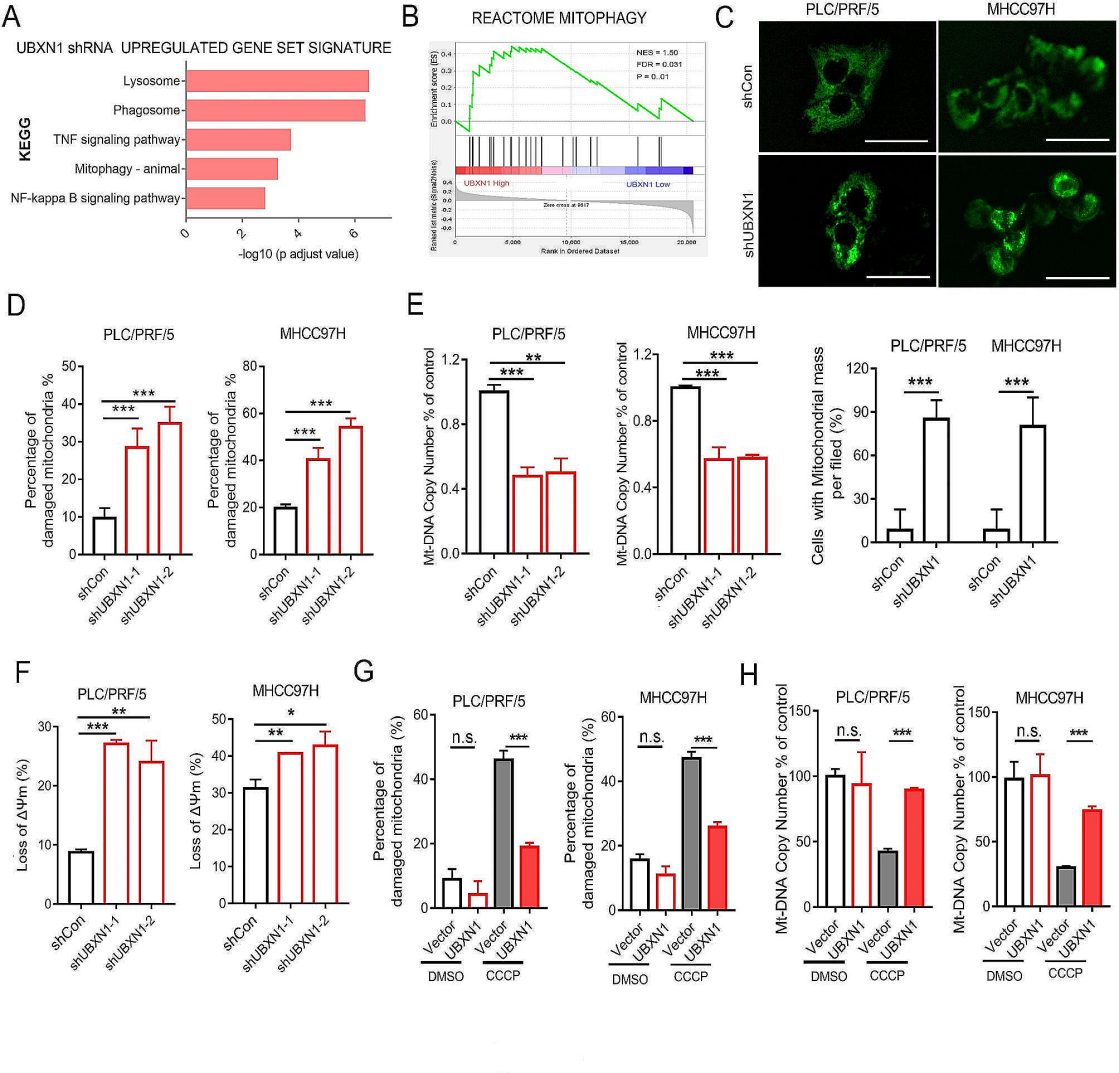
Inhibiting UBXN1 promotes mitochondrial damage in HCC cells. (**A**) KEGG enrichment analysis of UBXN1-upregulated genes in MHCC97H cells using compilation C5 (MSigDB). Upregulated genes were determined with the criteria: *p*<0.05 and log_2_ (fold change) ≥ 1. (**B**) GSEA of TCGA-LIHC dataset with *UBXN1* transcript levels and REACTOME_MITOPHAGY signature. (**C**) Representative pictures of mitochondrial morphology stained with MitoTracker Green in indicated PLC/PRF/5 and MHCC97H cells. Scale bar: 100 μm. (**D**) The indicated cells were stained with MitoTracker Deep Red (50 nM) for 15 min. The percentage of damaged mitochondria in HCC cells was measured by FACS. (**E**) mtDNA copy numbers in PLC/PRF/5 and MHCC97H cells were analyzed by qPCR. (**F**) The indicated cells were stained with JC-1 (5µM) for 15 min. The analysis of mitochondrial membrane potential loss was measured by FACS. (**G**) The indicated cells were treated with CCCP (20µM) for 12–24 h and stained with MitoTracker Deep Red (50 nM) for 15 min. The percentage of damaged mitochondria in HCC cells was measured by FACS. (**H**) mtDNA copy numbers analyzed by qPCR with tRNA-Leu to GAPDH in PLC/PRF/5 and MHCC97H cells with or without UBXN1 overexpression, CCCP (20µM) for 12–24 h. The data are presented as mean ± SD. *, *p <* 0.05; **, *p <* 0.01; ***, *p <* 0.001 by two-tailed Student t test

To investigate the detailed mechanisms underlying the pro-survival effects of UBXN1, we conducted Kyoto Encyclopedia of Genes and Genomes (KEGG) enrichment analysis of the differentially expressed genes (DEGs) in UBXN1-knockdown MHCC97H cells compared with control cells and found that the DEGs were notably enriched in signaling pathways related to mitochondrial homeostasis and NF-κB signaling (Fig. 4A). Accordingly, GSEA of the transcriptional profiles of the TCGA-LIHC cohort revealed that the mitophagy-related signatures were upregulated in the UBXN1^high^ tumor group (Fig. 4B). We also found a strong correlation between the mitophagy signature and the apoptosis signature in liver tumors from the TCGA-LIHC cohort (Supplementary 3 A-B). Next, we observed mitochondrial phenotypes and found that the knockdown of UBXN1 resulted in a significant increase in the number of HCC cells with abnormal mitochondrial aggregation (Fig. 4C). The MitoTracker Deep Red probe was then used to label intact mitochondria, and FACS analysis revealed that damaged mitochondria were robustly more abundant in UBXN1-knockdown cells than in control cells (Fig. 4D). In addition, a noticeable reduction in mtDNA copy number, accompanied by severe loss of membrane potential, was observed in PLC/PRF/5 and MHCC97H cells with UBXN1-knockdown (Fig. 4E-F). In contrast, upon treatment with CCCP, which blocks electron flow from PS II to PS I, ectopic expression of UBXN1 effectively inhibited mitochondrial damage, membrane potential reduction and cellular apoptosis in PLC/PRF/5 and MHCC97H cells (Fig. 4G-H, Supplementary 3 C-D), suggesting that UBXN1 is required for the maintenance of mitochondrial integrity under stress conditions. Next, to explore whether NF-κB signaling pathway was involved in UBXN1-regulated mitochondrial homeostasis, we first incubated HCC cells with CCCP, which induced mitochondria damage within 24 h in HCC cells (Fig. 4G), and found that the expression of active form of NF-κB (p65), p-p65, was comparable in HCC cells treated with CCCP for 0–24 h (supplementary Fig. 3E). Of note, UBXN1-knockdown was not capable to induce the expression of p-p65 in PLC/PRF/5 and LPC-HRas cells (supplementary Fig. 3F). These results therefore imply that UBXN1 inhibits the damage and loss of mitochondria in HCC cells independently of regulating NF-κB signaling pathway.

#### UBXN1 interacts with PHB and regulates its expression

**Fig. 5:**
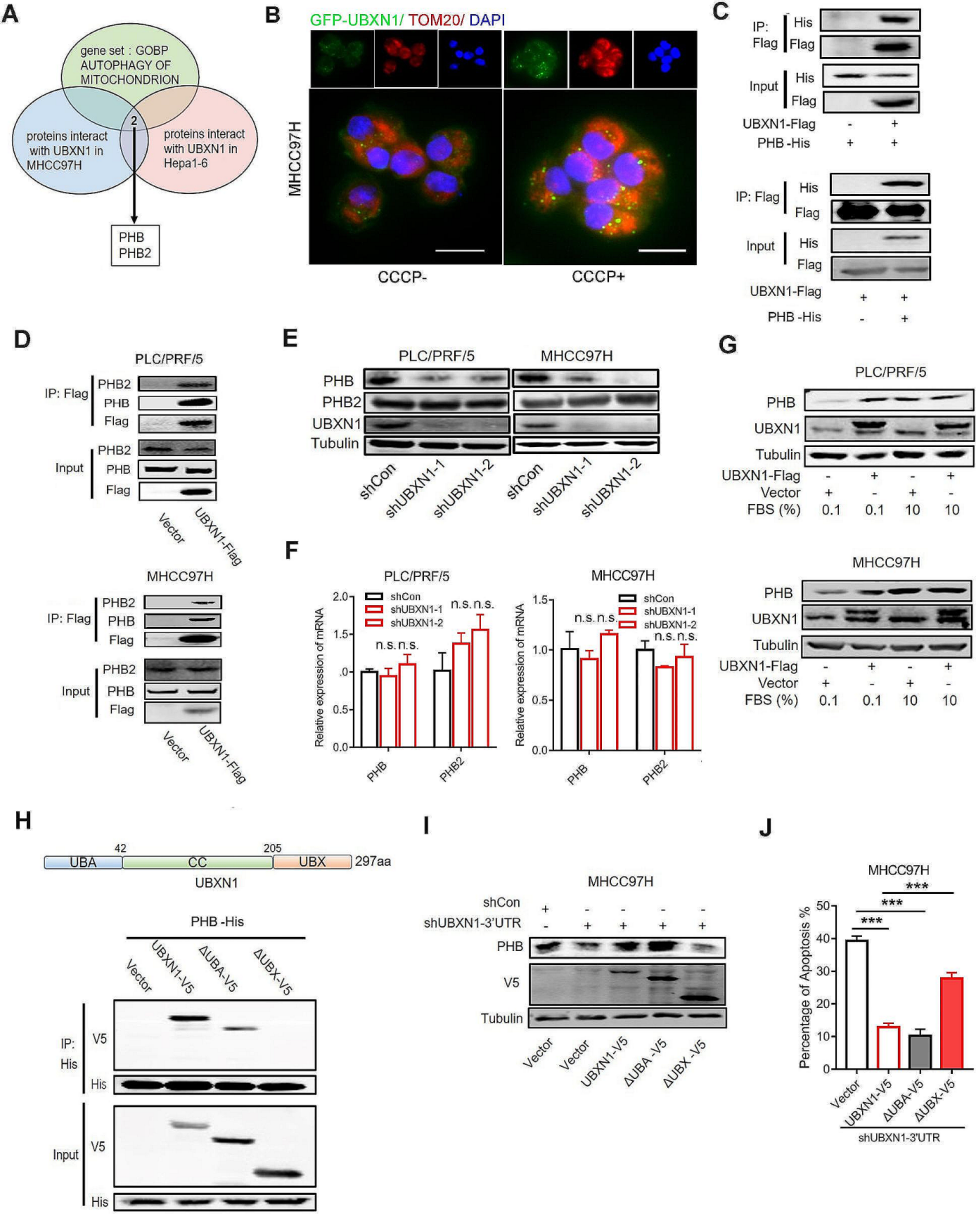
UBXN1 interacts with PHB and maintains its expression. (**A**) Venn diagram illustrated UBXN1 interactors (overlap based on IP-MS of MHCC97H and Hepa1-6, unique peptides > 2) with mitophagy regulator (GOBP AUTOPHAGY OF MITOCHONDRION). (**B**) GFP-UBXN1-expressed MHCC97H cells were treated with or without CCCP for 2 h MHCC97H cells. Representative pictures of IF staining of indicated cells was shown. (**C**) HEK293T cells were transient transfected with PHB-His together with UBXN1-Flag or control plasmid. Total lysates were immunoprecipitated with Flag M2 affinity gel after 48 h and the indicated proteins were detected by western blotting. (**D**) Total lysates from indicated PLC/PRF5 and MHCC97H cells were immunoprecipitated with Flag M2 affinity gel and then indicated proteins were detected by western blotting. (**E**) Western blotting analysis of indicted proteins in MHCC97H and PLC/PRF5 cells transduced with lentivirus *UBXN1* shRNA or shRNA-control plasmid. (**F**) qRT-PCR analysis of PHB and PHB2 in MHCC97H and PLC/PRF5 cells transduced with lentivirus *UBXN1* shRNA or shRNA-control plasmid. (**G**) Indicated PLC/PRF/5 and MHCC97H cells coculturing with or without medium containing 0.1% FBS for 24 h serum starvation. The expression of indicated proteins was measured by western blotting. (**H**) HEK293T cells were transient transfected with PHB-His together with UBXN1-V5, UBXN1-ΔUBA-V5, UBXN1-ΔUBX-V5 or control plasmid. Total lysates were immunoprecipitated with His affinity gel after 48 h and the indicated proteins were detected by western blotting. (**I**) Endogenous UBXN1 was knocked down by transfecting lentiviral shRNA targeting the 3′UTR of *UBXN1* in UBXN1-, UBXN1-ΔUBA- and UBXN1-ΔUBX-overexpressed MHCC97H cells. The indicated proteins were detected by western blotting. (**J**) Flow cytometry analysis of apoptotic ratio in indicated MHCC97H cells. The data are presented as mean ± SD. **, *p <* 0.01; ***, *p <* 0.001 by two-tailed Student t test

To investigate proteins involved in UBXN1-regulated mitochondrial homeostasis, we coimmunoprecipitated UBXN1 from human MHCC97H cells and mouse Hepa1-6 cells and performed mass spectrometry analysis to screen for UBXN1-interacting proteins. PHB and PHB2, which are present in the inner mitochondrial membrane, were identified as candidates (Fig. 5A). We further explored the subcellular localization of UBXN1 by transfecting HCC cells with a plasmid encoding the UBXN1-GFP fusion protein and observed that UBXN1 was mainly located and dispersed in the cytoplasm of cells cultured under normal conditions (Fig. 5B). Notably, the localization patterns of the UBXN1 protein changed, and the UBXN1 protein became more focally aggregated and colocalized with TOM20, a mitochondrial marker, in cells treated with CCCP (Fig. 5B), emphasizing the involvement of UBXN1 in the regulation of mitochondrial function. Then, we co-expressed UBXN1 with PHB or PHB2 in HEK293T cells and found that UBXN1 interacted with both PHB and PHB2 (Fig. 5C, Supplementary 4 A). Consistently, we also confirmed this interaction in the PLC/PRF/5 and MHCC97H HCC cell lines (Fig. 5D). Remarkably, we found that the level of PHB, but not the PHB2 protein, was decreased in UBXN1-knockdown HCC cells vs. control cells (Fig. 5E). Notably, knockdown of UBXN1 had little effect on the mRNA expression of both PHB and PHB2 (Fig. 5F). In addition, increased levels of PHB protein were detected in UBXN1-overexpressing cells under serum starvation conditions (Fig. 5G).

To explore the functional domain of UBXN1 in regulating PHB expression, we generated two truncated mutants of UBXN1, the ΔUBA mutant (with a 1–42 aa deletion) and the ΔUBX mutant (with a 205–313 aa deletion), to measure the ability of these mutants to bind PHB. We found that the UBX domain is essential for this interaction (Fig. 5H). Functionally, the overexpression of wild-type UBXN1 or the ΔUBA mutant, but not the ΔUBX mutant, robustly increased PHB expression and inhibited the cellular apoptosis induced by UBXN1-knockdown (Fig. 5I-J). In summary, these data suggest that a functional interaction between PHB and UBXN1 regulates tumor cell survival.

### UBXN1 function in promoting mitochondrial homeostasis in tumors depends on PHB

**Fig. 6:**
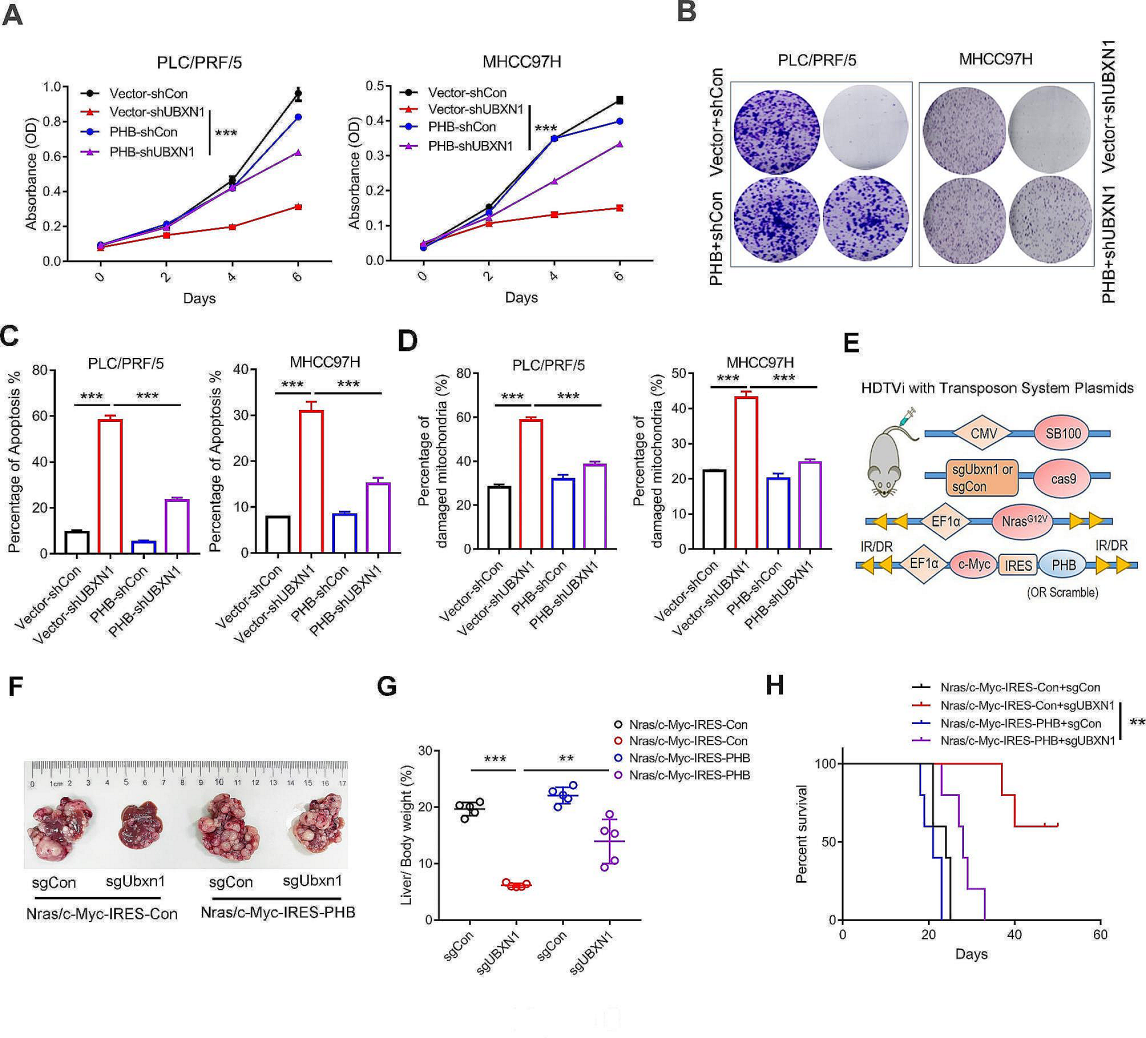
PHB counteracts UBXN1-knockdown-mediated HCC cell apoptosis and mitochondrial damage. (**A**–**B**) MTT assay (**A**) and colony formation assay (**B**) were used for measured proliferation of indicated cells. (**C**–**D**) Percentage of apoptotic cells (**C**) and damaged mitochondria (**D**) were measured by FACS. (**E**–**H**) The indicated vectors were hydrodynamic injected into C57BL/6 mice (*n* = 5). Schematic of vectors (**E**), morphology (**F**), ratio of liver/body weight (**G**) or Kaplan-Meier overall survival curves (**H**) were shown. Student’s t-test, ***p < 0.001

We next investigated the functional requirement of the PHB-UBXN1 interaction in liver tumorigenesis. We found that knockdown of PHB induced cell arrest, apoptosis, and severe mitochondrial damage (Supplementary 5 A–D), which reflects the importance of the PHB-UBXN1 interaction in maintaining mitochondrial homeostasis. Accordingly, ectopic expression of PHB significantly attenuated the cell proliferation arrest induced by UBXN1-knockdown (Fig. 6A-B, Supplementary 5E) and alleviated mitochondrial damage and the induction of cell apoptosis (Fig. 6C-D). More importantly, we integrated a CRISPR/*Cas9* deletion of *Ubxn1* into an *NRas*/*c-Myc*-driven liver tumor model with or without PHB overexpression (Fig. 6E). Although *Ubxn1* knockout effectively attenuated *NRas*/*c-Myc*-induced liver tumor growth (sg *Ubxn1*/*NRas*/*c-Myc*-IRES-Con vs. sg *Con*/*NRas*/*c-Myc*-IRES-Con), PHB expression significantly promoted liver tumorigenesis in the *Ubxn1*-knockout groups of mice (sg *Ubxn1*/*NRas*/*c-Myc*-IRES-PHB vs. sg *Ubxn1*/*NRas*/*c-Myc*-IRES-Con, Fig. 6F–H). Collectively, these results indicate that PHB is required for the UBXN1-mediated maintenance of mitochondrial homeostasis and liver tumorigenesis.

## Discussion

Tumor cells are adept at achieving a survival advantage by maintaining mitochondrial homeostasis upon exposure to endogenous or exogenous stresses [35–37]. The detailed mechanism by which mitochondrial homeostasis is maintained in tumor cells, however, remains elusive. Here, we identified UBXN1 as a tumor promoter in HCC via the maintenance of mitochondrial integrity. The expression of UBXN1 is aberrantly upregulated in human HCCs and mouse liver tumors, and high UBXN1 expression is associated with poor overall survival in patients with HCC. Mechanistically, the interaction between UBXN1 and PHB contributes to mitochondrial integrity, thereby promoting HCC cell survival and liver tumorigenesis.

UBXN1 is aberrantly expressed in several types of cancer and is implicated in the regulation of multiple biological processes through interactions with the multifunctional AAA-ATPase p97/VCP protein or ubiquitinated proteins via its UBX or UBA domain [38, 39]. Recent studies have demonstrated that the expression of UBXN1 is heterogeneous across tumors but generally decreased in gliomas [30]. Our results showed that the expression levels of UBXN1 are generally increased in the HCC samples examined. Interestingly, some of these samples exhibited marginal alterations or even decreased expression of UBXN1. Since the intertumor (tumor by tumor) heterogeneity in genetic background or biological context substantially exists in HCC [40], the compensatory mechanisms consisting of overlapping molecular events or alteration of the surrogates may be present and account for this divergence in UBXN1 expression. Nevertheless, our findings are the first to reveal that UBXN1 is highly expressed in HCC tissues and mouse liver tumors and contributes to HCC growth and malignant progression.

UBXN1 has diverse and sometimes opposite functions in regulating tumor progression due to differences in its substrates and cancer types [29–31]. YTHDF2 and PRADX decrease UBXN1 expression at the posttranscriptional level and thereby facilitate NF-κB signaling activity in glioblastoma and colon adenocarcinoma progression [30, 31]. In contrast, UBXN1 promotes AKT signaling and cell proliferation, migration and invasion in prostate cancer cells [29]. Our RNA-seq data revealed an increase in NF-κB signaling in UBXN1-knockdown MHCC97H cells, but silencing UBXN1 did not promote the expression of the active form of NF-κB in human PLC/PRF/5 or murine LPC-HRas HCC cells. Furthermore, mitochondrial damage induced by CCCP could not be sufficient to rapidly activate NF-κB signaling. Therefore, the promotion of liver tumorigenesis by UBXN1 is mainly due to the regulation of mitochondrial homeostasis but not NF-κB signaling.

Maintenance of mitochondrial homeostasis depends on mitochondrial quality control mechanisms that control mitochondrial integrity and function [41, 42]. A recent study showed that the inhibition of UBXN1 attenuates PINK1-mediated mitophagy via the recruitment of MFN2 to damaged mitochondria in cells treated with CCCP [27], emphasizing the role of UBXN1 in the clearance of damaged mitochondria. Similar to the findings of the present study, knockdown of UBXN1 triggered mitochondrial damage, resulting in excessive accumulation of dysfunctional mitochondria. Notably, we revealed that UBXN1 interacts with the mitochondrial inner membrane protein PHB and maintains its expression, which is required for mitochondrial membrane integrity. PHB and PHB2 are evolutionarily conserved scaffold proteins that form a multimeric ring complex at the inner mitochondrial membrane that acts as a protein and lipid scaffold to ensure the function of the inner mitochondrial membrane [43–45]. Consistent with our observation, PHB deficiency disrupts the integrity of the mitochondrial membrane and triggers NOXA-mediated apoptosis in multiple myeloma cells [22, 46]. Intriguingly, pharmacologically inhibiting PHB induces mitochondrial-dependent apoptosis and decreases the progression and development of gastric tumors [23, 47]. These findings suggest that PHB plays a prominent role in regulating UBXN1-mediated mitochondrial quality control and that PHB is a tumor-promoting factor in certain types of cancers.

Taken together, our findings revealed that UBXN1 hyperactivation frequently occurs and is crucial for liver tumorigenesis and that the tumor-promoting function of UBXN1 occurs through the UBXN1-PHB interaction, which regulates mitochondrial homeostasis.

### Electronic supplementary material

Below is the link to the electronic supplementary material.

Supplementary Material 1

## Data Availability

The RNA-seq data were deposited in the NCBI Gene Expression Omnibus under GEO: 247383.

## Abbreviations

HCC: Hepatocellular carcinoma
PHB: Prohibitin
mtDNA: mitochondrial DNA
ROS: reactive oxygen species
UBX: ubiquitin regulatory X
UBA: ubiquitin-associated domain
CCCP: Carbonyl Cyanide3-ChloroPhenylhydrazone
qRT-PCR: quantitative Real-Time Polymerase Chain Reaction
LC-MS/MS: Liquid chromatography-tandem mass spectrometry
GO: Gene Ontology
GSEA: Gene Set Enrichment Analysis
LIHC: Liver hepatocellular carcinoma
HDTVi: hydrodynamic tail-vein injection
SB: Sleeping Beauty
KEGG: Kyoto Encyclopedia of Genes and Genomes
Co-IP: Coimmunoprecipitation
