# Supplementary material for: UBXN1 promotes liver tumorigenesis by regulating mitochondrial homeostasis
Source: J Transl Med. 2024 May 21;22:485. doi: 10.1186/s12967-024-05208-5 (PMC11110256; doi:10.1186/s12967-024-05208-5)
Supplement: Supplementary file 1 — Supplementary Material 1 [file 12967_2024_5208_MOESM1_ESM.docx]

**UBXN1 promotes liver tumorigenesis by regulating mitochondrial homeostasis**

Kun Jiao ^a, b, 1^, Guiqin Xu ^b, 1^, Yun Liu ^b^, Zhaojuan Yang ^b^, Lvzhu Xiang ^b^, Zehong Chen ^b^, Chen Xu ^b^, You Zuo ^b^, Zhibai Wu ^b^, Ningqian Zheng ^b^, Wangjie Xu ^c^, Li Zhang ^b,^ **, and Yongzhong Liu ^a, b,^ *.

^a^ *State Key Laboratory of* *Systems Medicine for Cancer, Shanghai Cancer Institute, Renji Hospital, School of Biomedical Engineering, Shanghai Jiao Tong University, Shanghai, 200032, PR China*

^b^ *State Key Laboratory of Systems Medicine for Cancer, Shanghai Cancer Institute, Renji Hospital, School of Medicine, Shanghai Jiao Tong University, Shanghai, 200240, PR China*

*^c^ Laboratory Animal Center, Instrumental Analysis Center, Shanghai Jiao Tong University, Shanghai, 200240, China.*

1 Co-first author.

* Corresponding author.

** Corresponding author.

**Supplementary Information:**

1. Supplementary Figures S1~S5

2. Supplementary Tables 1~5


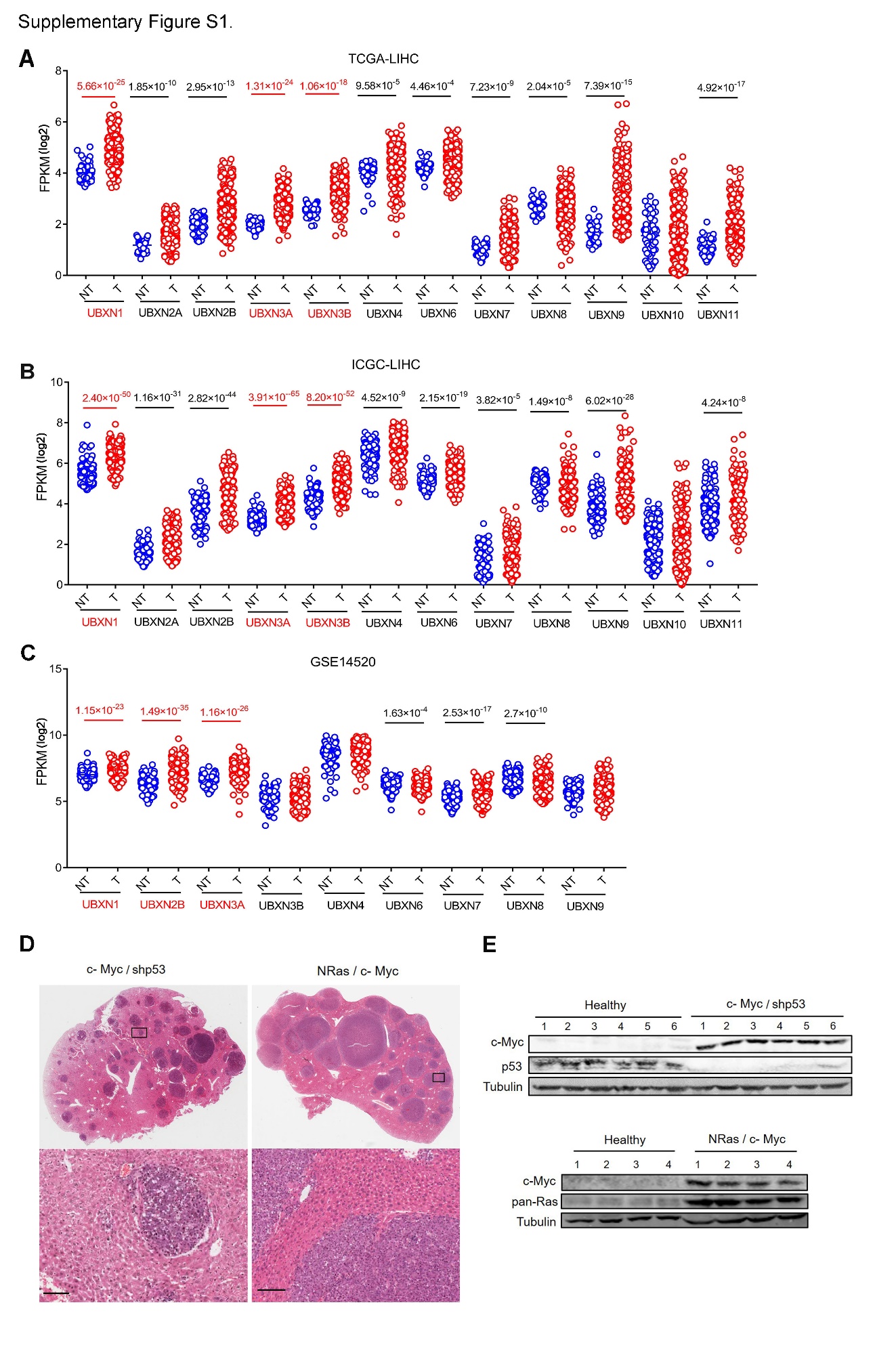


**Supplementary Figure S1.** (A-C) HCC samples derived from TCGA-LIHC (NT = 50, T = 374), ICGC-LIHC (NT = 202, T = 243) or GSE14520 (NT = 241, T = 247) databases were divided into two groups (NT VS T). The red fonts are the top 3 with the most significant differences in *p*-values. (D) H&E stain of mouse livers at 4 or 7 weeks post hydrodynamic injection related to Fig.1F. Scale bar: 50 μm. (E) Western blotting analysis of indicted proteins related to Fig.1F.


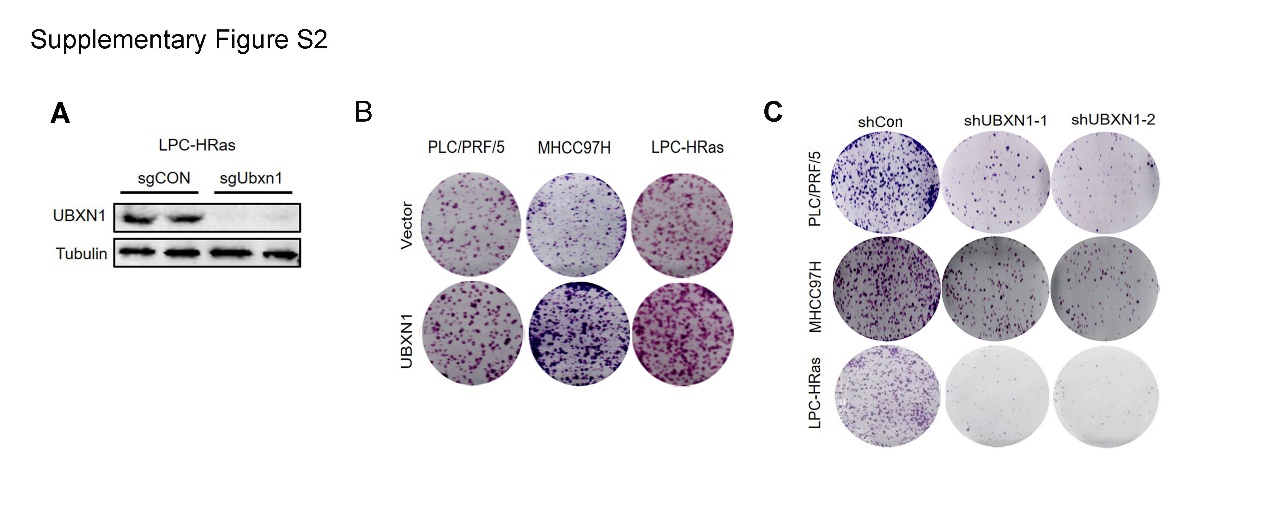


**Supplementary Figure S2.** (A) Western blotting analysis of indicated proteins in Ubxn1-knockout LPC-HRas cells. (B-C) The indicated cells were cultured with medium containing 0.1% FBS with starvation for one day or two, and then switched to complete medium for the following days. The growth capacity was tested by colony formation assay.


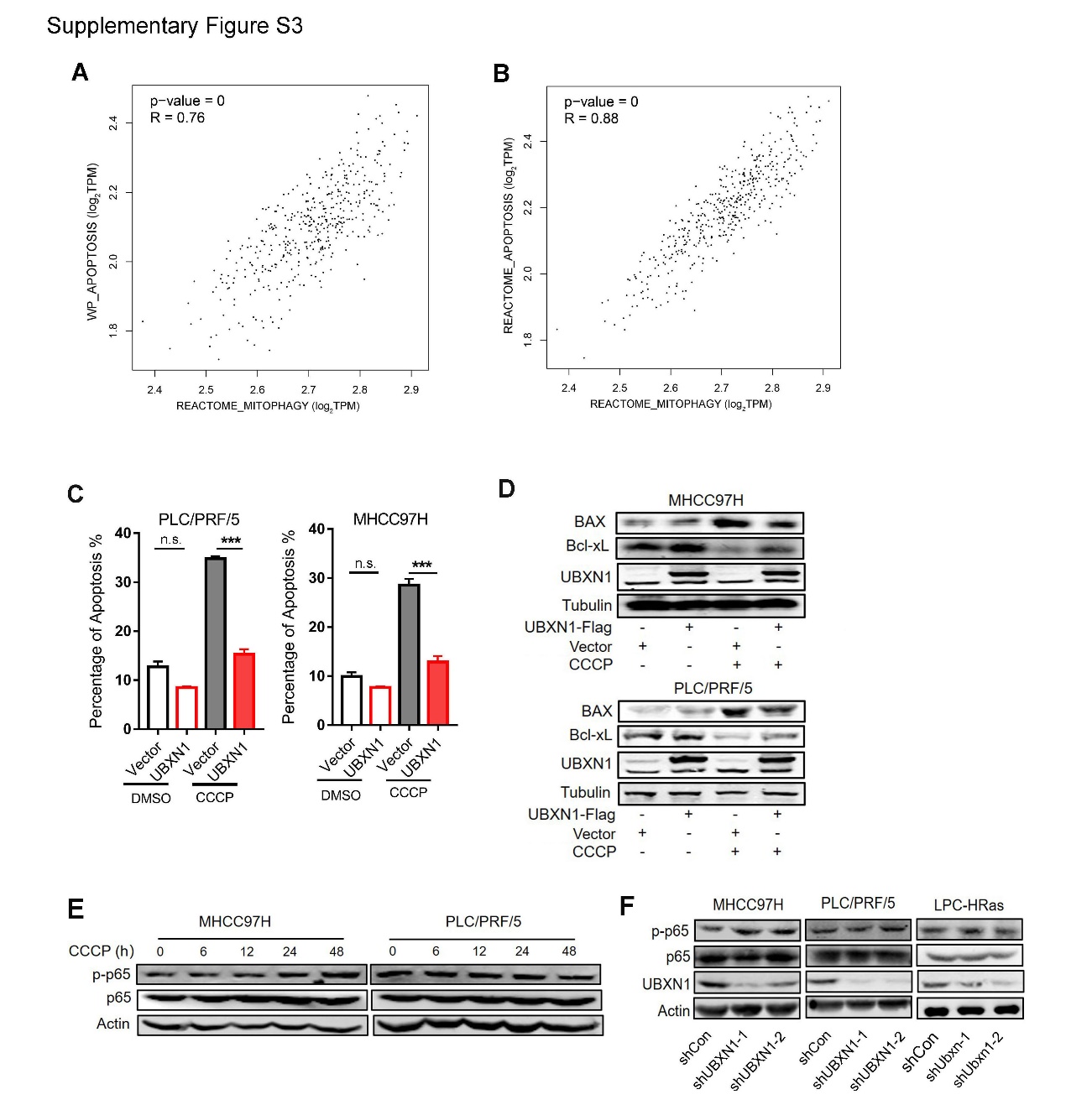


**Supplementary Figure S3.** (A-B) The Pearson correlation analysis between REACTOME MITOPHAGY and (A) REACTOME REGULATION OF APOPTOSIS or (B) WP APOPTOSIS signature based on TCGA-LIHC database. (C) Flow cytometry analysis of apoptosis ratio in UBXN1-overexpressed PLC/PRF/5 and MHCC97H cells with or without CCCP (20μM) treatment for 12h or 24h. (D) Western blotting analysis of indicated proteins in PLC/PRF/5 and MHCC97H cells treating with CCCP (20μM) for 12h-24h. (E) Western blotting analysis of indicated proteins in PLC/PRF/5 and MHCC97H cells treated with CCCP (20μM) for 6,12,24 or 48h. (F) Western blotting analysis of indicted proteins in Ubxn1-knockdown or control MHCC97H, PLC/PRF5 or LPC-HRas cells. Student’s t-test, ****p <* 0.001

**
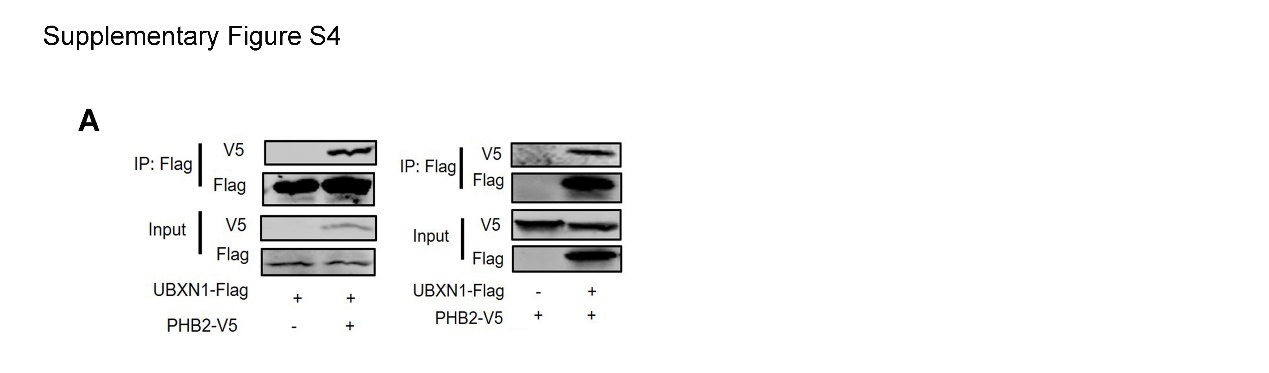
**

**Supplementary Figure S4.** (A) HEK293T cells were transient transfected with PHB2-V5 together with UBXN1-Flag or control plasmid. Total lysates were immunoprecipitated with Flag M2 affinity gel after 48h and the indicated proteins were detected by western blotting.


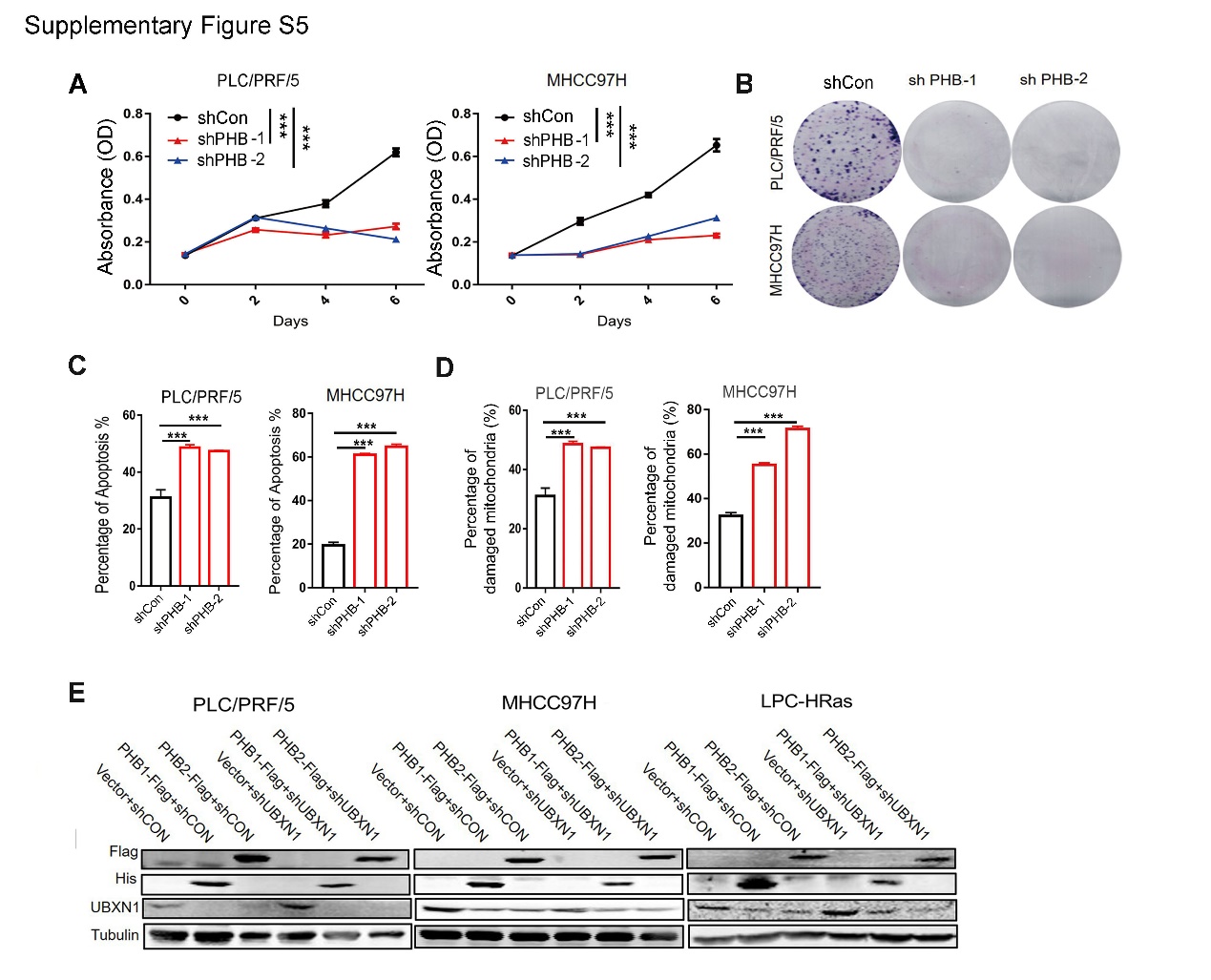


**Supplementary Figure S5.** (A-B) MTT assay (A) and colony formation assay (B) were used for measured proliferation of indicated cells. (C-D) Percentage of apoptotic cells (C) and damaged mitochondria (D) were measured by FACS. (E) PLC/PRF/5, MHCC97H and LPC-HRas cells transfected with indicated plasmids and then followed by UBXN1 shRNA or shRNA-control. Western blotting analysis for indicted proteins in indicated cells was measured. Student’s t-test, ****p <* 0.001

**Supplementary Table S1. Antibodies for IHC, immunofluorescence staining and immunoblotting**

| **Primary Antibodies** | **Company** | **Catalog Number** |
| --- | --- | --- |
| anti-UBXN1 | Proteintech | 16135-1-AP |
| anti-V5 | ABclonal | AE089 |
| anti-BAX | Proteintech | 50599-2-Ig |
| anti-Bcl-xL | ABclonal | A0209 |
| anti-cleaved-caspase3 | Cell Signaling Technology | 9664S |
| anti-PHB | Abmart | MA9268 |
| anti-α-Tubulin | Santa Cruz Biotechnology | sc-69969 |
| anti-β-Actin | Santa Cruz Biotechnology | sc-47778 |
| anti-Flag | Sigma-Aldrich | F1804 |
| anti-His | ABclonal | AE003 |
| anti-Flag | ABclonal | AE092 |
| anti-His | ABclonal | AE068 |
| anti-PHB2 | ABclonal | A4504 |
| anti-TOM20 | ABclonal | A6774 |
| anti-p53 | ABclonal | A21630 |
| anti-pan-RAS | ABclonal | A19779 |
| anti-c-Myc | ABclonal | A11394 |
| anti-p65 | Cell Signaling Technology | 3034S |
| anti-p-p65 | Cell Signaling Technology | 3033S |
| **Second Antibodies** | | |
| anti-mouse IRDye680RD | Li-COR | 926-68072 |
| anti-rabbit IRDye800CW | Li-COR | 926-32211 |
| anti-mouse IRDye800CW | Li-COR | 926-32210 |
| AlexaFluor555-labeled GoatAnti-Mouse IgG(H+L) | Beyotime | A0460 |

**Supplementary Table S2. Sequences for shRNA and sgRNA**

| shUBXN1-1# (human) | 5′-CCCGATGTGGACGAGCCTTTA -3′ |
| --- | --- |
| shUBXN1-2#(human) | 5′-AGGAGTTAGCAGCCAGACAAA -3′ |
| ShUBXN1-3#3’UTR (human) | 5′-GCACTGACATCTCCTTCCTAA-3′ |
| shUbxn1-1#(mouse) | 5′- GAGTATGACCAGTGTCGTATA-3′ |
| shUbxn1-2#(mouse) | 5′- CTGGAGAAAGCAGACCCATTT-3′ |
| sgUbxn1 (mouse) | 5′-CACAGGGAACCAGGGCATCG-3′ |
| shPHB-1# (human) | 5′-CCGTGGGTACAGAAACCAATT -3′ |
| shPHB-2#(human) | 5′-CGTGGTGAACTCTGCCTTATA -3′ |

**Supplementary Table S3. Primers for qPCR**

| **qPCR primers** | |
| --- | --- |
| human GAPDH-F | 5′-CATGAGAAGTATGACAACAGCCT-3′ |
| human GAPDH-R | 5′-AGTCCTTCCACGATACCAAAGT-3′ |
| human UBXN1-F | 5′-CGGGAGCGTGAAGAAAGAGA -3′ |
| human UBXN1-R | 5′- CCACACTGCCACCATACTTCT-3′ |
| human PHB-F | 5′- ACCACGTAATGTGCCAGTCA-3′ |
| human PHB-R | 5′- TAGTCCTCTCCGATGCTGGT-3′ |
| human PHB2-F | 5′- AATCCGTGTTCACCGTGGAAG-3′ |
| human PHB2-R | 5′- GGTCTGGCCCGAATGTCATA-3′ |
| mouse GAPDH-F | 5ʹ-AGGTCGGTGTGAACGGATTTG-3ʹ |
| mouse GAPDH-R | 5ʹ-TGTAGACCATGTAGTTGAGGTCA-3ʹ |
| mouse Ubxn1-F | 5ʹ-AGAACGAGAAAAGCAGCGGA-3ʹ |
| mouse Ubxn1-R | 5ʹ-TCTCGAACCCTTTGTCTGGC-3ʹ |

**Supplementary Table 4. Clinical information of HCC patients.**

| **Number** | **Gender** | **Age** | **Clinical Stage** |
| --- | --- | --- | --- |
| Case1 | Male | 63 | II |
| Case2 | Female | 51 | II |
| Case3 | Male | 56 | II |
| Case4 | Male | 38 | II |
| Case5 | Male | 50 | II |
| Case6 | Male | 57 | III |
| Case7 | Male | 63 | II |
| Case8 | Male | 60 | III |
| Case9 | Male | 74 | III |
| Case10 | Male | 37 | III |
| Case11 | Male | 57 | II |
| Case12 | Male | 56 | II |
| Case13 | Male | 66 | II |
| Case14 | Male | 63 | II |
| Case15 | Male | 56 | II |
| Case16 | Male | 55 | II |
| Case17 | Female | 31 | III |
| Case18 | Male | 53 | II |
| Case19 | Male | 51 | II-III |
| Case20 | Male | 57 | II |
| Case21 | Male | 63 | III |

**Supplementary Table 5. Clinical information of TMA.**

| **Number** | **type** | **age** | **gender** | **grade** |
| --- | --- | --- | --- | --- |
| A1 | cancerous | 55 | male | I-II |
| A2 | parcancerous |  |  |  |
| A3 | cancerous | 42 | male | I-II |
| A4 | parcancerous |  |  |  |
| A5 | cancerous | 55 | male | II |
| A6 | parcancerous |  |  |  |
| A7 | cancerous | 43 | male | II |
| A8 | parcancerous |  |  |  |
| A9 | cancerous | 64 | female | II |
| A10 | parcancerous |  |  |  |
| A11 | cancerous | 59 | male | II |
| A12 | parcancerous |  |  |  |
| A13 | cancerous | 43 | male | II |
| A14 | parcancerous |  |  |  |
| A15 | cancerous | 63 | male | II |
| A16 | parcancerous |  |  |  |
| B1 | cancerous | 56 | female | II |
| B2 | parcancerous |  |  |  |
| B3 | cancerous | 33 | male | II |
| B4 | parcancerous |  |  |  |
| B5 | cancerous | 48 | male | II |
| B6 | parcancerous |  |  |  |
| B7 | cancerous | 64 | female | II |
| B8 | parcancerous |  |  |  |
| B9 | cancerous | 60 | male | II |
| B10 | parcancerous |  |  |  |
| B11 | cancerous | 31 | male | II |
| B12 | parcancerous |  |  |  |
| B13 | cancerous | 39 | male | II |
| B14 | parcancerous |  |  |  |
| B15 | cancerous | 53 | female | II |
| B16 | parcancerous |  |  |  |
| C1 | cancerous | 45 | male | II |
| C2 | parcancerous |  |  |  |
| C3 | cancerous | 48 | male | II |
| C4 | parcancerous |  |  |  |
| C5 | cancerous | 53 | male | II |
| C6 | parcancerous |  |  |  |
| C7 | cancerous | 55 | male | II |
| C8 | parcancerous |  |  |  |
| C9 | cancerous | 48 | male | II |
| C10 | parcancerous |  |  |  |
| C11 | cancerous | 57 | male | II |
| C12 | parcancerous |  |  |  |
| C13 | cancerous | 38 | male | II |
| C14 | parcancerous |  |  |  |
| C15 | cancerous | 43 | male | II |
| C16 | parcancerous |  |  |  |
| D1 | cancerous | 65 | male | II |
| D2 | parcancerous |  |  |  |
| D3 | cancerous | 58 | male | II |
| D4 | parcancerous |  |  |  |
| D5 | cancerous | 54 | female | II |
| D6 | parcancerous |  |  |  |
| D7 | cancerous | 47 | male | II |
| D8 | parcancerous |  |  |  |
| D9 | cancerous | 52 | male | II-III |
| D10 | parcancerous |  |  |  |
| D11 | cancerous | 44 | male | II-III |
| D12 | parcancerous |  |  |  |
| D13 | cancerous | 56 | male | II-III |
| D14 | parcancerous |  |  |  |
| D15 | cancerous | 44 | male | II-III |
| D16 | parcancerous |  |  |  |
| E1 | cancerous | 73 | male | II-III |
| E2 | parcancerous |  |  |  |
| E3 | cancerous | 61 | male | II-III |
| E4 | parcancerous |  |  |  |
| E5 | cancerous | 42 | male | II-III |
| E6 | parcancerous |  |  |  |
| E7 | cancerous | 35 | male | II-III |
| E8 | parcancerous |  |  |  |
| E9 | cancerous | 55 | male | II-III |
| E10 | parcancerous |  |  |  |
| E11 | cancerous | 58 | male | II-III |
| E12 | parcancerous |  |  |  |
| E13 | cancerous | 47 | female | III |
| E14 | parcancerous |  |  |  |
| E15 | cancerous | 42 | male | III |
| E16 | parcancerous |  |  |  |
| F1 | cancerous | 43 | male | III |
| F2 | parcancerous |  |  |  |
| F3 | cancerous | 60 | male | III |
| F4 | parcancerous |  |  |  |
| F5 | cancerous | 42 | female | III |
| F6 | parcancerous |  |  |  |
| F7 | cancerous | 43 | male | III |
| F8 | parcancerous |  |  |  |
| F9 | cancerous | 45 | male | III |
| F10 | parcancerous |  |  |  |
| F11 | cancerous | 47 | male | III |
| F12 | parcancerous |  |  |  |
| F13 | cancerous | 40 | female | III |
| F14 | parcancerous |  |  |  |
| F15 | cancerous | 65 | male | III |
| F16 | parcancerous |  |  |  |
| G1 | cancerous | 54 | male | III |
| G2 | parcancerous |  |  |  |
| G3 | cancerous | 42 | male | III |
| G4 | parcancerous |  |  |  |
| G5 | cancerous | 50 | male | III |
| G6 | parcancerous |  |  |  |
| G7 | cancerous | 55 | male | III |
| G8 | parcancerous |  |  |  |
| G9 | cancerous | 45 | male | III |
| G10 | parcancerous |  |  |  |
| G11 | cancerous | 49 | female | III |
| G12 | parcancerous |  |  |  |
| G13 | cancerous | 44 | male | III |
| G14 | parcancerous |  |  |  |
| G15 | cancerous | 64 | female | III |
| G16 | parcancerous |  |  |  |
| H1 | cancerous | 58 | male | III |
| H2 | parcancerous |  |  |  |
| H3 | cancerous | 52 | male | III |
| H4 | parcancerous |  |  |  |
| H5 | cancerous | 57 | male | III |
| H6 | parcancerous |  |  |  |
| H7 | cancerous | 45 | male | III |
| H8 | parcancerous |  |  |  |
| H9 | cancerous | 53 | male | III |
| H10 | parcancerous |  |  |  |
| H11 | cancerous | 40 | male | III |
| H12 | parcancerous |  |  |  |
| H13 | cancerous | 36 | male | III |
| H14 | parcancerous |  |  |  |
| H15 | cancerous | 40 | male | III |
| H16 | parcancerous |  |  |  |
| I1 | cancerous | 45 | male | III |
| I2 | parcancerous |  |  |  |
| I3 | cancerous | 61 | female | III |
| I4 | parcancerous |  |  |  |
| I5 | cancerous | 53 | male | III |
| I6 | parcancerous |  |  |  |
| I7 | cancerous | 61 | male | III |
| I8 | parcancerous |  |  |  |
| I9 | cancerous | 74 | female | III |
| I10 | parcancerous |  |  |  |
| I11 | cancerous | 61 | female | III |
| I12 | parcancerous |  |  |  |
| I13 | cancerous | 74 | male | III |
| I14 | parcancerous |  |  |  |
| I15 | cancerous | 49 | female | III |
| I16 | parcancerous |  |  |  |
| J1 | cancerous | 67 | male | III |
| J2 | parcancerous |  |  |  |
| J3 | cancerous | 47 | male | III |
| J4 | parcancerous |  |  |  |
| J5 | cancerous | 58 | male | III |
| J6 | parcancerous |  |  |  |
| J7 | cancerous | 64 | male | III |
| J8 | parcancerous |  |  |  |
| J9 | cancerous | 68 | male | III |
| J10 | parcancerous |  |  |  |
| J11 | cancerous | 54 | male | III - IV |
| J12 | parcancerous |  |  |  |
| J13 | cancerous | 35 | male | IV |
| J14 | parcancerous |  |  |  |
| J15 | cancerous | 44 | male | IV |
| J16 | parcancerous |  |  |  |
